# Ecosystem engineering by foxes is mediated by the landscape context—A case study from steppic burial mounds

**DOI:** 10.1002/ece3.4224

**Published:** 2018-06-22

**Authors:** Laura Godó, Béla Tóthmérész, Orsolya Valkó, Katalin Tóth, Réka Kiss, Szilvia Radócz, András Kelemen, Péter Török, Eva Švamberková, Balázs Deák

**Affiliations:** ^1^ Department of Ecology University of Debrecen Debrecen Hungary; ^2^ MTA‐DE Biodiversity and Ecosystem Services Research Group Debrecen Hungary; ^3^ MTA's Post‐Doctoral Research Programme Debrecen Hungary; ^4^ MTA‐DE Lendület Functional and Restoration Ecology Research Group Debrecen Hungary; ^5^ Department of Botany University of South Bohemia in České Budějovice České Budějovice Czech Republic

**Keywords:** biodiversity, disturbance, fragmentation, isolation, kurgan, sacred sites, steppe, weeds

## Abstract

In intensively used landscapes, remnant grassland fragments are often restricted to places unsuitable for agricultural cultivation. Such refuges are the ancient burial mounds called “kurgans,” which are typical landscape elements of the Eurasian steppe and forest steppe zone. Due to their hill‐like shape, loose soil structure and undisturbed status kurgans provide proper habitats for burrowing mammals. Accordingly, grassland vegetation on kurgans is often exposed to bioturbation, which can influence the habitat structure and plant species pool. In our study, we explored the effect of fox burrows and landscape context on the habitat properties and vegetation composition of small landscape elements, using kurgans as model habitats. We surveyed the vegetation of fox burrows and that of the surrounding grassland on five kurgans situated in cleared landscapes surrounded by arable lands and five kurgans in complex landscapes surrounded by grazed grasslands. We recorded the percentage cover of vascular plants, the amount of litter, and soil moisture content in twelve 0.5 m × 0.5 m plots per kurgan, in a total of 120 plots. We found that foxes considerably transformed habitat conditions and created microhabitats by changing the soil nutrient availability and reducing total vegetation cover and litter. Several grassland specialist species, mostly grasses (*Agropyron cristatum*,* Elymus hispidus,* and *Stipa capillata*) established in the newly created microhabitats, although the cover of noxious species was also considerable. We found that landscape context influenced the sort of species which could establish on kurgans by affecting the available species pool and soil moisture. Our results revealed that foxes act as ecosystem engineers on kurgans by transforming abiotic and biotic conditions by burrowing. Their engineering activity maintains disturbance‐dependent components of dry grasslands and increases local environmental heterogeneity.

## 1 INTRODUCTION

European dry grasslands are among the most species‐rich habitats in the temperate zone and harbor many rare and endangered species (Dengler, Janišová, Török, & Wellstein, 2014). The landscape‐level loss of the habitats has led to the fragmentation and isolation of the remaining grassland patches by a matrix unsuitable for the existence of grassland species (Lindborg, Plue, Andersson, & Cousins, 2014; Martín‐Queller, Albert, Dumas, & Saatkamp, 2017). As a consequence of landscape transformations, the proportion of complex landscapes harboring a high proportion of seminatural habitats has decreased considerably. In parallel, there has been an increase in the extension of cleared landscapes which are characterized by a high proportion of man‐made habitats and the presence of only a few isolated seminatural habitat patches (Tscharntke et al., 2012). European lowland dry grasslands with fertile soils have been especially threatened by landscape transformation, as their soil is especially suitable for intensive agricultural utilization (Cousins, 2006; Deák, Tóthmérész et al., 2016; Lindborg et al., 2012). In cleared landscapes, plant populations of the remnant grassland patches are especially affected by unpredictable environmental events, disturbances, and demographic stochasticity (Heinken & Weber, 2013). The instability of isolated grassland patches is also due to the limited seed rain because of the absence of grasslands in the landscape and the lack of dispersal vectors (Lindborg et al., 2012; Poschlod & Wallis De Vries, 2002).

Seminatural dry grasslands have been maintained by extensive management measures such as grazing or mowing, which eliminate the accumulated biomass and decrease inter‐ and intraspecific competition (Tälle et al., 2016). While grassland management by grazing or mowing is a feasible option in complex landscapes, in cleared landscapes conservational management of small habitat patches is often impossible due to the lack of financial resources and difficulties in logistics (Deák, Valkó, Török, & Tóthmérész, 2016; Valkó et al., 2018). Thus, due to the cessation of management, kurgans cutoff from surrounding grasslands are often prone to litter accumulation, shrub encroachment, and the spread of competitor species (Deák, Tóthmérész et al., 2016; Sudnik‐Wójcikowska, Moysiyenko, Zachwatowicz, & Jabłońska, 2011). Altered habitat conditions together with the microsite limitation may lower the survival and establishment rates of specialist species (Gazol et al., 2012).

Against all odds, small grassland patches located in cleared landscapes, such as midfield islands, channel dikes, and roadside verges often harbor a high biodiversity of grassland species (Fekete et al., 2017; Sudnik‐Wójcikowska et al., 2011). In the western part of the steppe biome (involving Hungary, Romania, Bulgaria, Ukraine, and the European parts of Russia), the several hundred thousand ancient burial mounds called “kurgans” also play a crucial role in preserving grassland vegetation, especially in intensively used agricultural landscapes (Deák, Tóthmérész et al., 2016; Dembicz et al., 2016; Tóth, Pethe, & Hatházi, 2014). Even though grasslands on kurgans can be considered stable habitats which are usually characterized by closed grass‐dominated vegetation, they are often exposed to several types of human and natural disturbances (Deák, Tóthmérész et al., 2016). The latter mostly involve the activity of burrowing mammals, which can act as ecosystem engineers on kurgans (Sudnik‐Wójcikowska & Moysiyenko, 2008).

Ecosystem engineers by definition are organisms that can directly or indirectly modulate the availability of resources for other species, by making physical changes in their biotic or abiotic environment. Ecosystem engineer species modify, maintain, and create habitats (Fischer et al., 2017; Jones, 2012). Soil mixing by burrowing animals is an important component of pedogenesis, increasing habitat heterogeneity even on large spatial scales, and thus an important driver of vegetation dynamics (Wilkinson, Richards, & Humphreys, 2009). Soil disturbance by small mammals can provide a proper microsite for the establishment of grassland specialist plant species, but can also cause habitat degradation and create establishment gaps for weeds (Jones, 2012; Kurek, Kapusta, & Holeksa, 2014; Müller et al., 2014; Zimmermann et al., 2014).

In Europe, the red fox (*Vulpes vulpes* Linnaeus, 1758) is one of the most widespread mammals that makes considerable habitat changes by its burrowing activity. Foxes prefer to burrow on slopes near open areas (Uraguchi & Takahashi, 1998) and avoid settling in areas with a high human presence, due to centuries of persecution (Pagh, 2008). Kurgans provide proper habitats for foxes, as they are elevated points in open landscapes and their soil mostly has a loose, loamy structure suitable for burrowing (Deák, Valkó et al., 2016). The activity of foxes results in an increased area of open microsites and also changes in the soil properties. The accumulating excrement and remnants of the prey can lead to an increase in the soil nutrient content near to the burrows (Macdonald, 1979; Monclús, Arroyo, Valencia, & de Miguel, 2009). Both the increased level of open soil surface and soil nutrient content can affect the vegetation recovery on abandoned fox burrows. Altered environmental conditions affect both the species richness and the abundance of specialist plants and species indicating degradation of the habitat (hereafter referred to as “noxious species”; Blackshaw et al., 2003). Moreover, foxes can also alter vegetation composition by zoochorous seed dispersal (Kurek et al., 2014).

We studied the effects of fox burrowing on the habitat properties and vegetation composition of grasslands on kurgans in cleared and complex landscapes. Our study questions were the following: (a) How does burrowing affect the habitat properties (soil moisture, soil nutrient content, and the amount of litter)? (b) Do the abandoned fox burrows support the establishment of grassland species or rather act as hotspots for the encroachment of noxious species? (c) Is the vegetation of fox burrows affected by the landscape context?

## 2 MATERIAL AND METHODS

### 2.1 Study sites

The studied kurgans are located in the Hortobágy National Park, Great Hungarian Plain, East Hungary (N 47°34′, E 21°9′). The region is characterized by a continental climate with a mean annual precipitation of 550 mm and a mean annual temperature of 9.5°C with high interannual fluctuations (Deák et al., 2014). The elevation ranges between 88 and 102 m a.s.l. The area is characterized by a mosaic of vast seminatural habitats and agricultural lands (Deák et al., 2014). The core area of the national park consists of extended complex landscapes with open habitats such as alkaline and loess grasslands, wetlands, and alkaline marshes. Its peripheries, lying outside the protected area, are characterized by cleared landscapes, which have been heavily affected by agriculture over the past centuries (Deák, Tóthmérész et al., 2016).

### 2.2 Selection criteria, description of the selected kurgans

For our study, we considered kurgans covered by seminatural dry grassland vegetation (alkali and loess grasslands). For the site selection, we used our own database containing localities and basic land cover data of 548 kurgans situated in the northern part of the Great Hungarian Plain. The location and land cover data of the kurgans were derived from topographical maps and satellite images and were partly collected during other research (see Deák, Valkó et al., 2016). We studied abandoned fox burrows where, in the absence of continuous disturbance, plant establishment could start. We omitted burrows with visible signs of recent fox activity such as fresh footprints, droppings, remaining parts of prey, and fresh digging.

We selected ten kurgans covered by grassland vegetation and harboring fox burrows. Five kurgans were embedded in a complex landscape, and five kurgans were situated in a cleared landscape (see Tscharntke et al., 2012; Supporting Information Appendices S1 and S2). Kurgans surrounded by more than 20% dry grasslands and a low proportion of arable lands within a radius of 200 m were categorized as “kurgans in a complex landscape.” Kurgans in a complex landscape had a connection with grasslands, thus they were grazed by cattle. Kurgans surrounded by more than 80% of croplands were categorized as “kurgans in a cleared landscape.” Kurgans in a cleared landscape were not connected to other grassland habitats and thus were not managed (Supporting Information Appendix S3). Due to the common agricultural practice of the region, kurgans in cleared landscapes were mostly situated in areas with fertile soils (mostly chernosemic soil) and characterized by loess grasslands, while kurgans in complex landscapes were situated in alkali landscapes characterized by alkali soils with a humus‐rich upper layer. To control for the possible consequences of the differences in the species pool of the grasslands with different landscape contexts, we mostly focused on the differences in the ecological indicator values and in the proportion of the functional groups. The studied kurgans were not affected by any human disturbance (building, plowing, and burning). The average height of the kurgans was 4.5 ± 0.7 m, and their average diameter was 43.4 ± 4.7 m.

### 2.3 Field sampling

On each kurgan we selected two subsites, with each subsite covering one fox burrow and the adjacent surrounding intact grassland. In each subsite, we designated three 50 × 50 cm sized plots on the surface of the fox burrow and three plots in the adjacent dry grassland which were not affected by the foxes (e.g., digging, trampling). The aspect and the inclination of the plots within each subsite was the same, with the grassland plots located 1–1.5 m from the edge of the burrow (Supporting Information Appendix S4). On each kurgan, we surveyed a total of 12 plots (6/subsite). Thus, in total we used 120 plots for the calculations. We recorded the percentage cover of all vascular plant species and the thickness and percentage cover of the litter layer in each plot in June 2017. We expressed the amount of litter by its volume calculated from the litter cover and thickness. We measured the volumetric moisture content of the upper 20 cm of the soil at three random points of each plot using a FieldScout TDR 300 soil moisture meter (resolution 0.1%). The area of seminatural dry grasslands and seminatural habitats in a buffer zone of 200 m around the kurgans was calculated using habitat maps compiled during the vegetation sampling. For the habitat maps, we used satellite images provided by the OpenLayers plugin of the Quantum GIS 2.2 (QGIS Development Team, 2009).

### 2.4 Data processing

For the statistical analyses, we categorized the species into two ecological groups: grassland specialists and noxious species (species indicating the degradation of the habitat). We considered herbaceous species of Festuco‐Brometea and Puccinellio‐Salicornea phytosociological classes as grassland specialists (Borhidi, 1995). We used the categories of the social behavior type system (Borhidi, 1995) for the classification of noxious species. Adventive competitors (AC), ruderal competitors (RC), and weeds (W) were considered noxious species. Species were assigned to four functional groups based on the simplified life form categories: perennial graminoids, perennial forbs, short‐lived graminoids, and short‐lived forbs. We obtained Ellenberg ecological indicator values of the species and calculated cover‐weighted scores on a plot level for water (WB) and nutrients (NB) adapted to the Hungarian conditions (Borhidi, 1995). For the classification of the species, see Supporting Information Appendix S5.

For visualizing the vegetation patterns, we used detrended correspondence analysis (DCA) based on specific cover scores. We used the soil moisture scores, the amount of litter, and the cover‐weighted ecological indicators values (WB, NB) as an overlay for the ordination (CANOCO 5; Ter Braak & Šmilauer, 2012). Indicator species analysis was used to identify species typical of the two studied microhabitat types (burrow and grassland) on kurgans situated in complex and cleared landscapes (Dufrêne & Legendre, 1997). For the analyses, we used the “labdsv” package in an R environment (R Core Team 2010, Roberts, 2010).

To explore the effects of burrowing and the landscape context on the amount of litter, soil moisture, and vegetation characteristics we used GLMMs (generalized linear mixed model; Zuur, Ieno, Walker, Saveliev, & Smith, 2009). Predictors included the microhabitat type (vegetation of fox burrow or undisturbed grassland), the landscape context (complex and cleared landscape), and their interaction. “Subsites nested in site” was included as a random factor to control for the possibility that the vegetation of plots from the same subsite and site are more similar to each other than expected by chance. For the calculations, we used plot level data (*n* = 120). Response variables were soil moisture, cover‐weighted ecological indicator values, amount of litter, total vegetation cover, species richness, and percentage cover of grassland specialists, noxious species, perennial forbs, perennial graminoids, short‐lived forbs, and short‐lived graminoids. Percentage cover scores were log‐transformed (log(*x* + 1)) to approximate them to normal distribution. We used normal distribution with an identity link for the calculations for the amount of litter, soil moisture scores, percentage cover scores, and cover‐weighted ecological indicator values. We used Poisson distribution with a log link for species richness scores. Fitted models were checked by plotting residuals and fitted values and predictors using Q‐Q plots (Zuur et al., 2009). Pairwise comparisons were calculated using Fisher's least significant difference (LSD) method. The analyses were performed using the SPSS 20 program.

## 3 RESULTS

**Figure 1:**
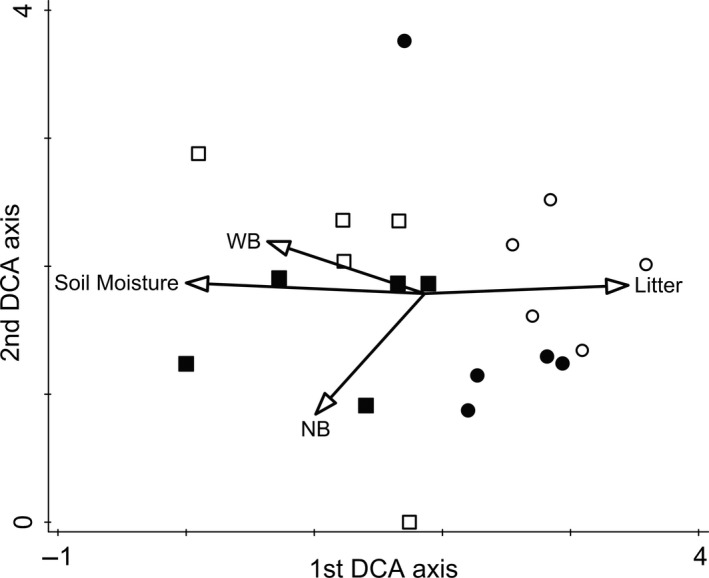
Detrended correspondence analysis plot of the environmental factors and study sites based on the species composition. Amount of litter, soil moisture, and cover‐weighted scores of Ellenberg ecological indicator values for water (WB), and nutrients (NB) were included as an overlay. Notations: filled symbols—fox burrows; empty symbols—adjacent grassland; circles—isolated kurgans; squares—nonisolated kurgans. Eigenvalues for the first and second axes were 0.488 and 0.355, respectively. The cumulative explained variances of the first and the second axes were 12.97% and 22.40%, respectively

We found altogether 127 vascular plant species in the surveyed plots, of which 38 were grassland specialist species (Supporting Information Appendix S5). We found several grassland specialists (such as *Carex praecox*,* Elymus hispidus*,* Euphorbia cyparissias*,* Phlomis tuberosa*,* Podospermum canum*,* Salvia nemorosa,* and *Thymus glabrescens*) in the grassland plots of kurgans in cleared landscapes. Indicator species of fox burrows in cleared landscapes were *Carduus acanthoides*,* Cynodon dactylon*,* Fallopia convolvulus*,* Papaver rhoeas,* and *Phragmites australis*. In grassland plots in complex landscapes *Achillea collina*,* Alopecurus pratensis*,* Convolvulus arvensis*,* Cruciata pedemontana*,* Galium verum*,* Poa angustifolia,* and *Trifolium striatum* were typical. Fox burrows in complex landscapes harbored mostly ruderals (such as *Bromus mollis*,* Bromus tectorum*,* Capsella bursa‐pastoris*,* Chenopodium album*,* Polygonum aviculare,* and *Torilis arvensis*; Supporting Information Appendix S6), but also several grassland specialist grasses (such as *Agropyron cristatum*,* E. hispidus,* and *Stipa capillata*) and some perennial specialist forbs (*Falcaria vulgaris, Salvia austriaca*) also occurred there (Supporting Information Appendix S6). The vegetation composition of fox burrow plots and grassland plots; as well as the vegetation of the plots from kurgans in cleared and complex landscapes were well separated by the DCA ordination (Figure 1). Plots from kurgans in complex landscapes were more scattered than the ones in cleared landscapes.

**Figure 2:**
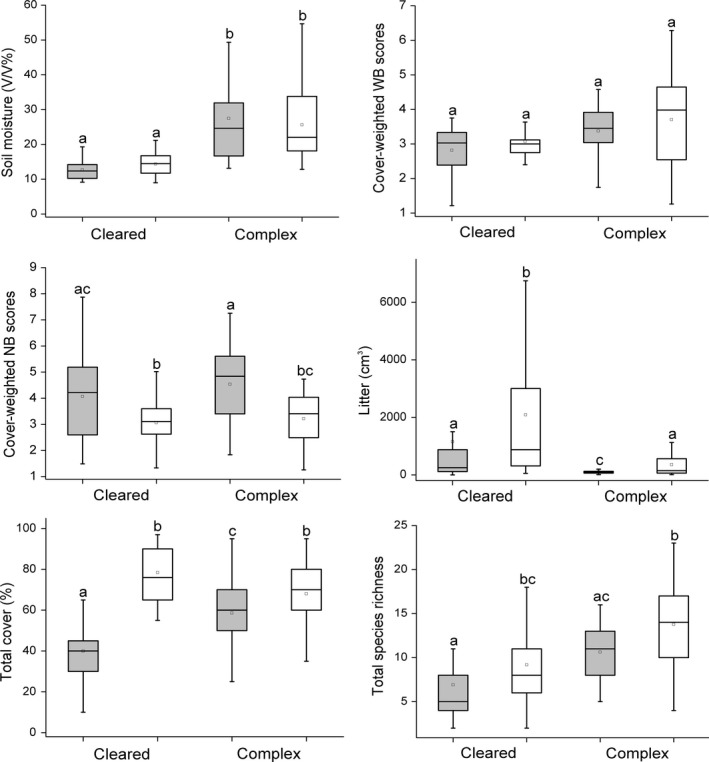
Soil moisture, cover‐weighted WB (ecological indicator value for soil moisture), and NB (ecological indicator value for nutrient content) scores, amount of litter, total vegetation cover, and total species richness on kurgans situated in cleared and complex landscapes. Gray boxes—fox burrows; white boxes—adjacent grasslands. The boxes show the interquartile range, the lower whiskers show the minimum, the upper whiskers show the maximum, and the inner lines display the median values. Superscript letters denote significant differences between groups (LSD test, *p *<* *0.05; *n* = 120)

Soils of kurgans embedded in cleared landscapes were characterized by a lower volumetric moisture content of the upper 20 cm than soils of kurgans in complex landscapes (coefficient ± *SE* = −0.219 ± 0.054; *t* = −4.072; *p* = 0.001). We did not observe differences in the cover‐weighted WB scores. High cover‐weighted NB scores were typical of the burrows (coefficient ± *SE* = 0.114 ± 0.025; *t* = 4.626; *p* = 0.001). The litter amount was lower on burrows than in the grassland plots (coefficient ± *SE* = −0.306 ± 0.122; *t* = −2.514; *p* = 0.013). Kurgans in cleared landscapes were characterized by a higher amount of litter compared to the ones situated in cleared landscapes (coefficient ± *SE* = 0.718 ± 0.213; *t* = 3.366; *p* = 0.001; Figure 2).

Total vegetation cover was significantly lower on burrows than in the grassland plots (coefficient ± *SE* = −0.072 ± 0.032; *t* = −2.246; *p* = 0.027); this pattern was more pronounced in cleared landscapes (coefficient ± *SE* = −0.250 ± 0.045; *t* = −5.499; *p* = 0.001). Kurgans in complex landscapes harbored significantly more species than those in cleared landscapes (coefficient ± *SE* = −0.479 ± 0.188; *t* = −2.549; *p* = 0.012). Total species richness was lower on fox burrows than in the grasslands, regardless of the landscape context (coefficient ± *SE* = −0.258 ± 0.075; *t* = −3.465; *p* = 0.001; Figure 2).

**Figure 3:**
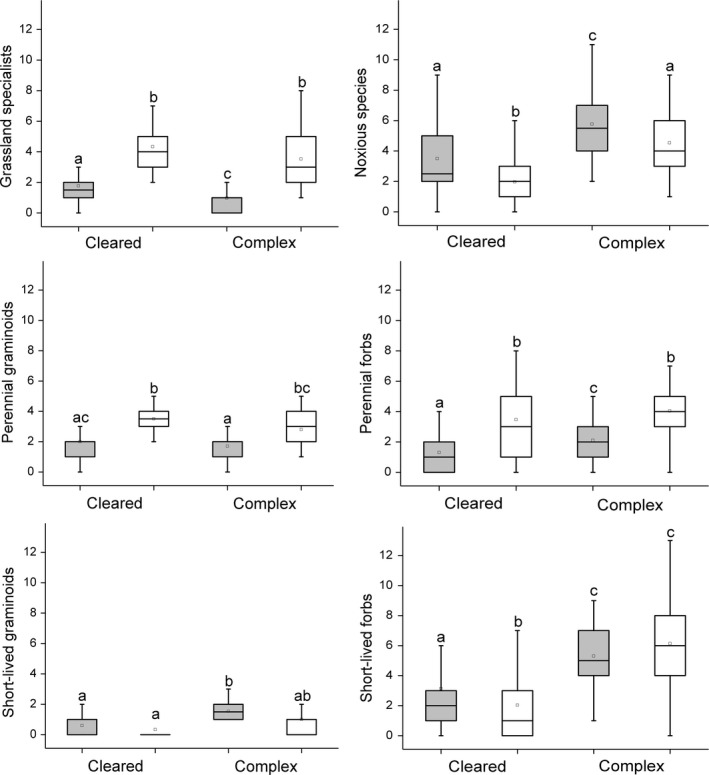
Species richness of ecological and functional species groups on kurgans situated in cleared and complex landscapes. Gray boxes—fox burrows; white boxes—adjacent grasslands. The boxes show the interquartile range, the lower whiskers show the minimum, the upper whiskers show the maximum, and the inner lines display the median values. Superscript letters denote significant differences between groups (LSD test, *p *<* *0.05; *n* = 120)

**Figure 4:**
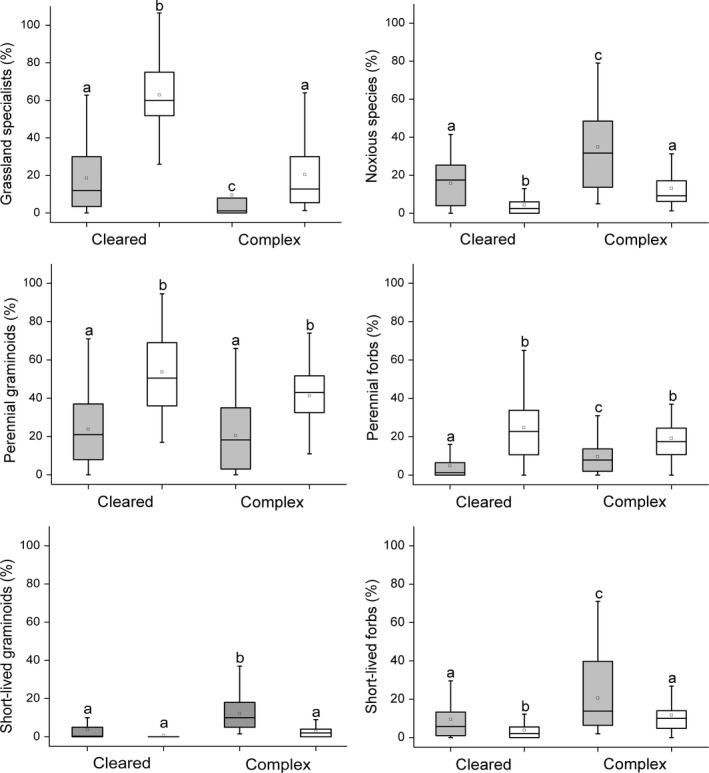
Percentage cover scores of ecological and functional species groups on kurgans situated in cleared and complex landscapes. Gray boxes—fox burrows; white boxes—adjacent grasslands. The boxes show the interquartile range, the lower whiskers show the minimum, the upper whiskers show the maximum, and the inner lines display the median values. Superscript letters denote significant differences between groups (LSD test, *p *<* *0.05; *n* = 120)

Species richness and cover of grassland specialists were significantly lower on the fox burrows (coefficient ± *SE* = −1.296 ± 0.210; *t* = −6.185; *p* = 0.001; coefficient ± *SE* = −0.568 ± 0.099; *t* = −5.726; *p* = 0.001; Figures 3 and 4). Cover of grassland specialists were higher in cleared landscapes than in complex landscapes (coefficient ± *SE* = 0.622 ± 0.167; *t* = 3.723; *p* = 0.001). Species richness and the cover of noxious species were higher on the burrows than in the grassland plots (coefficient ± *SE* = 0.241 ± 0.115; *t* = 2.100; *p* = 0.038; coefficient ± *SE* = 0.411 ± 0.073; *t* = 5.621; *p* = 0.001). Species richness and the cover of noxious species were lower on kurgans in cleared landscapes compared to those in complex landscapes (coefficient ± *SE* = −0.910 ± 0.247; *t* = −3.678; *p* = 0.001; coefficient ± *SE* = −0.480 ± 0.141; *t* = −3.409; *p* = 0.001). Species richness and cover of perennial forbs and graminoids were lower on the burrows (perennial forbs: coefficient ± *SE* = −0.653 ± 0.155; *t* = −4.201; *p* = 0.001; coefficient ± *SE* = −0.329 ± 0.105; *t* = −3.134; *p* = 0.002; perennial graminoids: coefficient ± *SE* = −0.499 ± 0.178; *t* = −2.811; *p* = 0.006; coefficient ± *SE* = −0.537 ± 0.097; *t* = −5.532; *p* = 0.001). Cover of short‐lived forbs and graminoids were higher on burrows (coefficient ± *SE* = 0.217 ± 0.092; *t* = 2.364; *p* = 0.020; coefficient ± *SE* = 0.573 ± 0.084; *t* = 6.818; *p* = 0.001). Cover of short‐lived forbs and graminoids (coefficient ± *SE* = −0.481 ± 0.143; *t* = −3.365; *p* = 0.001; coefficient ± *SE* = −0.341 ± 0.103; *t* = −3.296; *p* = 0.001) and the species richness of short‐lived forbs were lower in cleared landscapes (coefficient ± *SE* = −1.370 ± 0.335; *t* = −4.087; *p* = 0.001). In cleared landscapes the species richness of short‐lived forbs was higher on fox burrows than in the grassland plots (interaction; coefficient ± *SE* = 0.578 ± 0.197; *t* = 2.938; *p* = 0.004). In complex landscapes cover of short‐lived graminoids was higher on fox burrows compared to the grassland (coefficient ± *SE* = −0.314 ± 0.119; *t* = −2.645; *p* = 0.009), but there were no differences in kurgans embedded in a cleared landscape.

## 4 DISCUSSION

### 4.1 Habitat conditions

Our findings showed that foxes changed the habitat conditions on kurgans by creating microhabitats for plant establishment. Soil moisture was the same on the burrows and in the grassland vegetation. Guo, Mou, Jones, and Mitchell (2002) reported that soil moisture has rapid dynamics after soil disturbance. By bringing up moist soil layers, and due to the enhanced water infiltration, soil disturbances (such as burrowing) temporarily increase the soil moisture in the upper layers (see also Zhang, Zhang, & Liu, 2003). Later on, by evaporation it decreases to the predisturbance level in a short time. We found that soil moisture was lower on kurgans situated in cleared landscapes compared to those in complex landscapes. This is partly due to the unfavorable water holding capacity of arable lands (Schwartz, Evett, & Unger, 2003). Another reason might be that cleared landscapes were characterized by higher lying chernosemic soils, while complex landscapes were characterized by lower lying alkali soils. This pattern was also confirmed by the DCA ordination. Differences in volumetric soil moisture content were not confirmed by the WB scores. In spite of the moister habitat conditions on kurgans situated in complex landscapes, the proportion of the species typical of moist habitats was not significantly higher here. This can be explained by the “vigor hypothesis” which assumes that grazers generally opt for the most vigorous species with broad leaves and a high nutrient content. In our studied kurgans, these were the species characterized by high WB scores (Price, 1991; Rutter, 2006). Selective grazing considerably decreased their cover and therefore the cover‐weighted WB scores, as well.

We found a high proportion of nutrient‐demanding species, mostly noxious species such as *Amaranthus retroflexus*,* Arctium lappa*,* Carduus acanthoides*,* Hyoscyamus niger,* and *Onopordum acanthium* on fox burrows (Supporting Information Appendix S6). The presence of nutrient‐demanding species on the burrows can be explained by the confounding effect of the changes in the amount of soil nutrients and the high availability of open microsites around the burrows. The nutrient content of the soil might be changed by the excrement of the foxes and also by taking deeper soil layers to the surface of the kurgan (Kurek et al., 2014; Monclús et al., 2009). In contrast with grasslands on flat areas where the deeper soil layers (bedrock) generally contain less nutrients and humus, on kurgans the deeper layers have a relatively high nutrient and humus content (Deák, Tóthmérész et al., 2016). The reason for this is that kurgans were made from the topsoil and sod excavated from the neighboring areas (Lisetskii, Goleusov, Moysiyenko, & Sudnik‐Wójcikowska, 2014). As the effect of nutrient enrichment and disturbance on the vegetation composition cannot be clearly separated, we should assume that increased proportions of nutrient‐demanding species on burrows were at least partly due to the recent soil disturbance (Deák, Hüse, & Tóthmérész, 2016).

The decreased amount of litter on fox burrows was likely a direct outcome of the decreased biomass production in the recovering vegetation (also shown by the scores of total vegetation cover; Figure 1). Due to the lack of management, which could have removed the accumulated biomass, the amount of litter was significantly higher on kurgans located in a cleared landscape (see also Deák, Tóthmérész et al., 2016; Godó et al., 2017; Tälle et al., 2016). In contrast, on kurgans situated in complex landscapes, biomass was decreased by grazing, and also by the trampling of livestock, which contributes to the degradation of litter (Shariff, Biondini, & Grygiel, 1994).

### 4.2 Burrows and landscape context as drivers of vegetation composition

We found that total species richness was lower on the burrows than in the grasslands. It was also lower in cleared, compared to complex landscapes. Recent disturbance by foxes acted as an establishment filter for numerous taxa, and this effect was further influenced by the landscape context. In cleared landscapes, probably the limited availability of the species that could cope with the special habitat conditions provided by the kurgans might result in the reduced total species richness (Lavorel, McIntyre, Landsberg, & Forbes, 1997). On the one hand, due to the intensive agricultural use, the adjacent arable lands are extremely species poor, and on the other hand, the seed transport from the few neighboring species‐rich grassland patches is hindered by the lack of dispersal vectors. Contrary, in complex landscapes, there is an enhanced seed dispersal by grazing animals, which can transport high quantities of seeds of both grassland specialists and noxious species to the open microsites on their fur and in their droppings (Metera, Sakowski, Słoniewski, & Romanowicz, 2010; Poschlod & Wallis De Vries, 2002). Even though foxes can also act as dispersal vectors, their role is subordinate compared to megaherbivores. Furthermore, their role might be different in simple and complex landscapes (Kurek et al., 2014). They presumably disperse mainly seeds of zoochorous grassland specialist plant species in complex landscapes and mainly seeds of noxious species in simple landscapes. Foxes can also support plant establishment by taking buried seeds to the surface (Jalloq, 1975).

After disturbance, the chance of successful colonization by specialist species is usually low as specialists are not able to outcompete noxious species adapted to ruderal, nutrient‐rich conditions (Marvier, Kareiva, & Neubert, 2004; Valkó et al., 2017). We also found that disturbance by foxes decreased both the species richness and cover of those specialist species which are not adapted to a high level of habitat transformation (Grime, 1979; Figures 3 and 4, Supporting Information Appendices S5 and S6). The low recolonization rate of specialists was also due to their predominantly transient and low‐density seed bank, which likely considerably hampered their re‐establishment (Thompson, Bakker, & Bekker, 1997). Interestingly, we found a higher cover of grassland specialists on kurgans embedded in cleared landscapes than on those situated in complex landscapes. The reason for this might be due to the drier habitat conditions in isolated grassland fragments. Isolated dry grassland fragments in cleared landscapes are generally characterized by drier habitat conditions compared to the adjacent areas (Cousins, 2006; Lindborg et al., 2014). Dry habitat conditions favor xerophilous steppe specialists and suppress most of the noxious species on kurgans embedded in cleared landscapes (Deák, Valkó et al., 2016). This is especially true for kurgans which harbor grassland fragments with drier habitat conditions than the surrounding plain areas.

While disturbance by foxes suppressed most of the specialist perennial forbs (such as *Cruciata pedemontana, Euphorbia* spp., *Phlomis tuberosa*,* Podospermum canum, Salvia verticillata, Trifolium* spp*.,* and *Thymus glabrescens*), a certain sort of grassland specialist grasses (such as *Agropyron cristatum*,* Elymus hispidus,* and *Stipa capillata*) and perennial specialist forbs (*Falcaria vulgaris* and *Salvia austriaca*) could effectively establish on the disturbed soil surfaces. We found that tussock‐forming grass species were particularly successful in recolonization, due to their robust physiognomy and high competitive ability (Janeček, Janečková, & Lepš, 2007). This suggests that despite the recent disturbance causing a temporal local encroachment of noxious species, patches of disturbed surfaces can be overgrown by specialist species (Deák, Tóthmérész et al., 2016).

Open microsites provided by the burrows supported the encroachment of noxious species due to the decreased level of competition (Whitford & Kay, 1999), increased or changed nutrient availability (Kurek et al., 2014; Müller et al., 2014) and the reduced amount of litter (Foster & Gross, 1998; Xiong & Nilsson, 1999). A lack of interspecific competition, mostly by removing perennial tussock‐forming grasses, such as *Alopecurus pratensis* and *Festuca* spp., allowed the encroachment of several, mostly annual noxious species with a low competitive ability. Noxious species, especially the annual r‐strategists, could rapidly emerge from their persistent seed banks and the seed rain from the adjacent areas and could thus gain dominance in the newly established open microsites (Renne & Tracy, 2013). A decreased amount of litter on burrows also supported the establishment of noxious species due to the increased availability of light and the lack of any physical barrier effect of litter (Deák et al., 2011). The weed suppression effect of litter can be observed in kurgans situated in cleared landscapes. Burrows in cleared landscapes were characterized by a greater amount of litter than those in complex landscapes, and in parallel, the species number and cover of noxious species were lower compared to complex landscapes.

We found that in spite of the high propagule pressure potentially coming from the neighboring arable land (Sudnik‐Wójcikowska & Moysiyenko, 2010), there was a smaller proportion of noxious species in the grassland plots of cleared landscapes than in those of complex ones. In the grassland vegetation of the kurgans embedded in cleared landscapes, establishment of noxious species was hindered, likely due to the lack of open microsites and the presence of grassland species with a high competitive ability (Bullock, Hill, Silvertown, & Sutton, 1995; Deák, Valkó et al., 2016; Sudnik‐Wójcikowska et al., 2011). In complex landscapes, besides the disturbances associated with grazing (biomass removal and trampling), grazing animals could support the establishment of noxious species by epi‐ and endozoochory (Hulme, 2002).

The species pool of noxious species was different in cleared and in complex landscapes. In cleared landscapes, burrow plots were characterized by arable weeds (*Papaver rhoeas* and *Fallopia convolvulus*) and terrestrial reed (*Phragmites australis*), which are indicators of abandonment. In complex landscapes, there were several grazing‐tolerant species in the vegetation typical of pastures (*Elymus repens, Bromus mollis, B. tectorum, Hordeum murinum,* and *Polygonum aviculare*). Besides the landscape context, the occurrence of these species also reflects the different soil conditions.

Our research showed that red foxes act as ecosystem engineers on kurgans, as they change the vegetation composition and the structure of the community by transforming the habitat conditions and creating microhabitats for plant establishment. Foxes had a complex effect on the vegetation by changing the soil nutrient availability and reducing the accumulated litter on their burrows. Even though these disturbed patches were predominantly characterized by noxious species, they also provided opportunities for the recruitment of specialist species. By affecting the available species pool for re‐establishment, the landscape context significantly influenced the vegetation composition of burrows. Through burrowing, red foxes might function as ecosystem engineers. They maintain the disturbance‐dependent components of plant communities and increase local environmental heterogeneity.

## CONFLICT OF INTEREST

None declared.

## AUTHOR CONTRIBUTIONS

BD, LG, BT, and OV designed the experiment; LG, KT, SR, RK, ES, AK, OV, and BD collected data; BD, OV, and BT analyzed the data, BD, LG, BT, and OV wrote the manuscript with contributions from all coauthors.
