# Supplementary material for: Ecosystem engineering by foxes is mediated by the landscape context—A case study from steppic burial mounds
Source: Ecol Evol. 2018 Jun 22;8(14):7044–54. doi: 10.1002/ece3.4224 (PMC6065349; doi:10.1002/ece3.4224)
Supplement: Supplementary file 2 [file ECE3-8-7044-s002.docx]

**
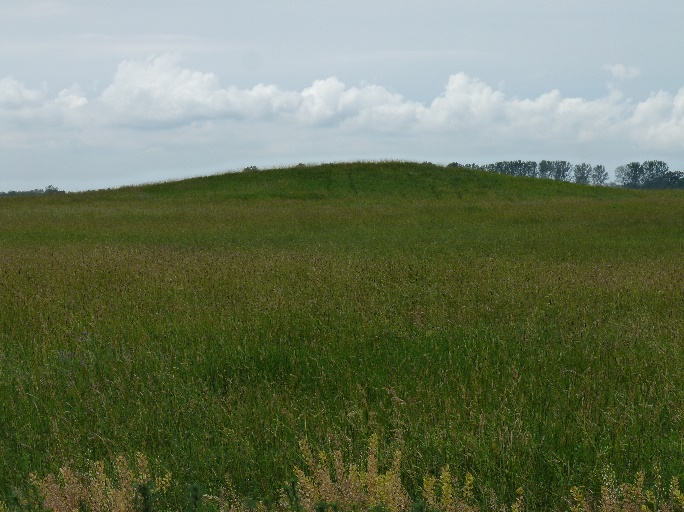

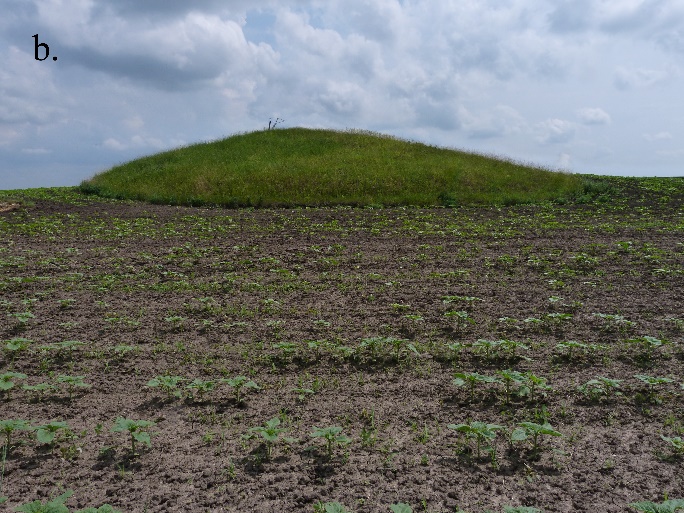
**

**A**

**B**


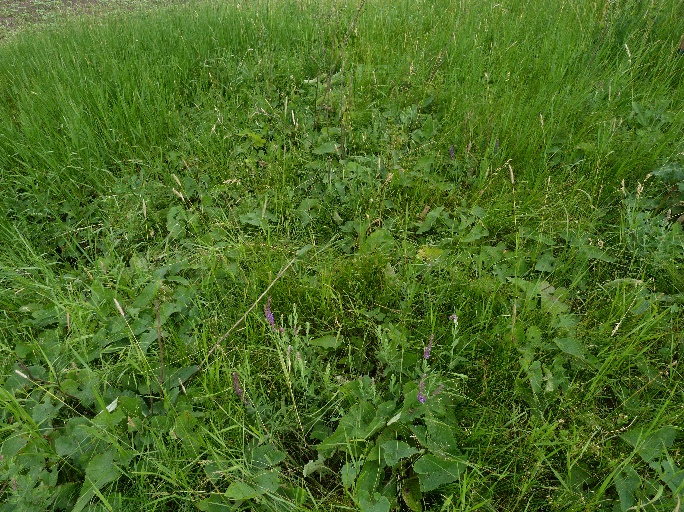
 **
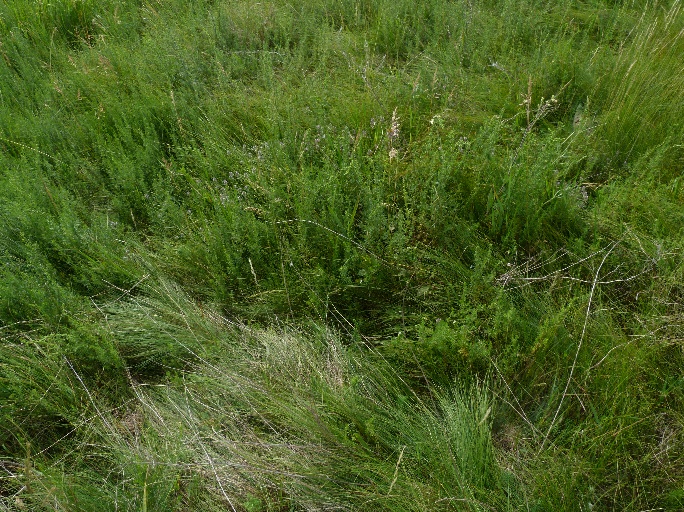
**

**C**

**D**


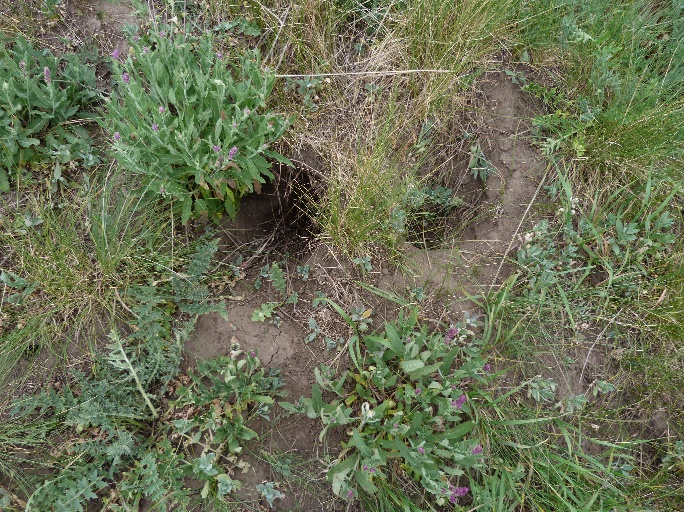

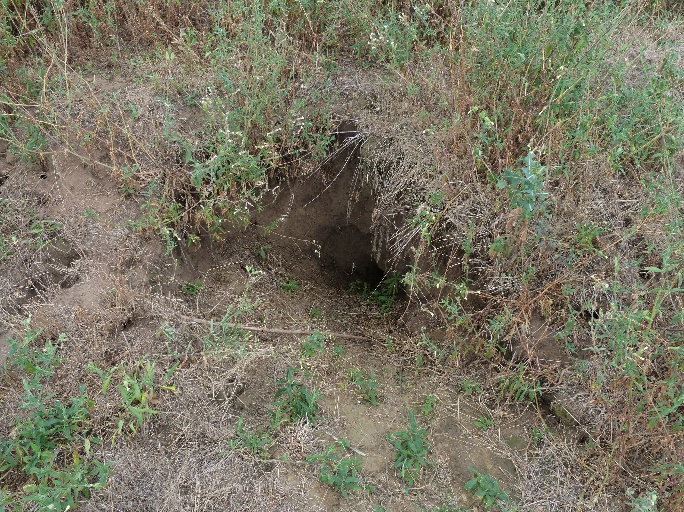


**E**

**F**

**Appendix 2.** Kurgans with steppe vegetation in the Hortobágy National Park, East-Hungary. A, the Kacskó-kurgan surrounded by grassland vegetation; B, Sólyom-kurgan embedded in a sunflower field; C-D, steppe vegetation with *Phlomis tuberosa*, *Galium verum*, *Carex praecox*, *Thymus glabrescens*; E, regeneration of specialist species (*Agropyron cristatum* and *Salvia nemorosa*) on fox burrow; F encroachment of weed species (*Melandrium album*) on fox burrow. Photos by B. Deák.
