# Supplementary material for: Ecosystem engineering by foxes is mediated by the landscape context—A case study from steppic burial mounds
Source: Ecol Evol. 2018 Jun 22;8(14):7044–54. doi: 10.1002/ece3.4224 (PMC6065349; doi:10.1002/ece3.4224)
Supplement: Supplementary file 3 [file ECE3-8-7044-s003.docx]

**Appendix 3.** Descriptive data on the landscape composition of kurgans embedded in cleared and complex landscapes. Percentage cover of semi-natural dry grasslands and all semi-natural habitats was calculated in the 200 m vicinity of the studied kurgans.

|  | **Percentage of semi-natural dry grasslands (%±SE)** | **Range** | | **Percentage of semi-natural habitats (%±SE)** | **Range** | |
| --- | --- | --- | --- | --- | --- | --- |
|  |  | **Min.** | **Max.** |  | **Min.** | **Max.** |
| Cleared | 8.34 ± 0.84 | 2.70 | 17.40 | 9.8 ± 0.76 | 3.00 | 17.40 |
| Complex | 42.02 ± 2.52 | 23.90 | 74.70 | 67.56 ± 2.55 | 41.30 | 99.80 |
