# Supplementary figures and images for: Ecosystem engineering by foxes is mediated by the landscape context—A case study from steppic burial mounds
Source: Ecol Evol. 2018 Jun 22;8(14):7044–54. doi: 10.1002/ece3.4224 (PMC6065349; doi:10.1002/ece3.4224)

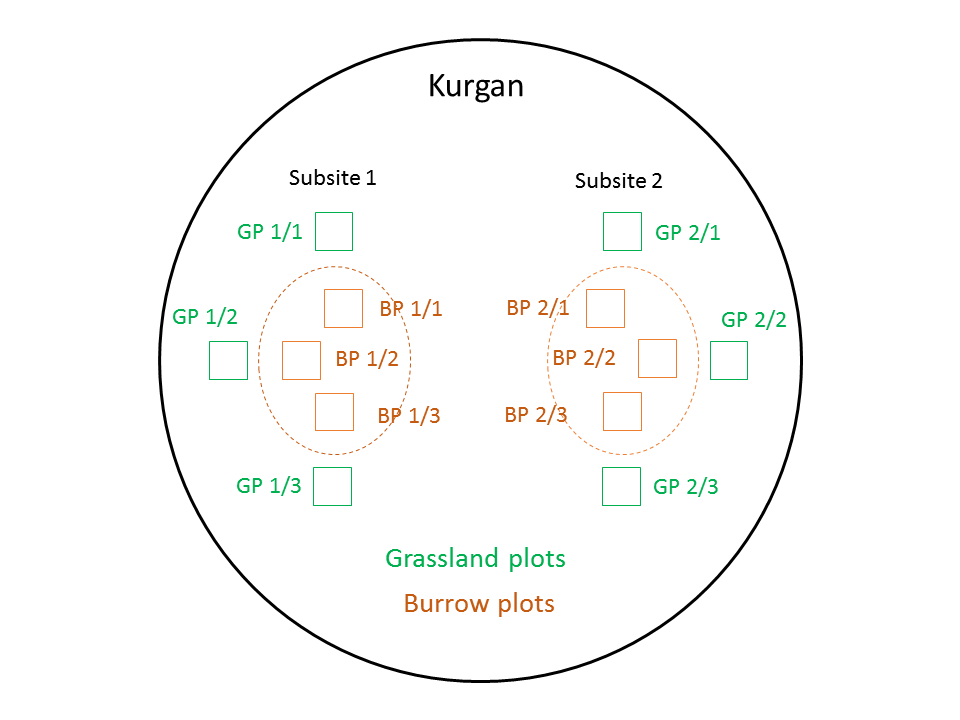


**Appendix 4.** Scheme of the sampling design.

Supplement: Supplementary file 4 [file ECE3-8-7044-s004.docx]
