# Supplementary material for: Ecosystem engineering by foxes is mediated by the landscape context—A case study from steppic burial mounds
Source: Ecol Evol. 2018 Jun 22;8(14):7044–54. doi: 10.1002/ece3.4224 (PMC6065349; doi:10.1002/ece3.4224)
Supplement: Supplementary file 6 [file ECE3-8-7044-s006.docx]

**Appendix 6.** Significant indicator species of fox burrows and intact grasslands on kurgans embedded in cleared and complex landscapes. Notations: IndVal – Indicator value; Freq – Frequency; *** *p*< 0.001; ** *p*< 0.01; **p*< 0.05.

| **Species** | **Landscape** | **Microhabitatat** | **IndVal**  **value** | ***p*** | **Freq** |
| --- | --- | --- | --- | --- | --- |
| *Poa angustifolia* | complex | grassland | 0.44 | *** | 61 |
| *Alopecurus pratensis* | complex | grassland | 0.41 | *** | 37 |
| *Convolvulus arvensis* | complex | grassland | 0.40 | *** | 55 |
| *Cruciata pedemontana* | complex | grassland | 0.37 | *** | 11 |
| *Achillea collina* | complex | grassland | 0.34 | *** | 21 |
| *Trifolium striatum* | complex | grassland | 0.33 | *** | 10 |
| *Galium verum* | complex | grassland | 0.31 | ** | 31 |
| *Cerastium dubium* | complex | grassland | 0.31 | *** | 20 |
| *Trifolium retusum* | complex | grassland | 0.30 | *** | 9 |
| *Taraxacum officinale* | complex | grassland | 0.23 | *** | 7 |
| *Podospermum canum* | complex | grassland | 0.23 | *** | 8 |
| *Potentilla argentea* | complex | grassland | 0.19 | ** | 15 |
| *Crepis rhoeadifolia* | complex | grassland | 0.16 | ** | 6 |
| *Stellaria graminea* | complex | grassland | 0.16 | ** | 6 |
| *Plantago lanceolata* | complex | grassland | 0.16 | ** | 7 |
| *Trifolium angulatum* | complex | grassland | 0.13 | * | 4 |
| *Cerastium semidecandrum* | complex | grassland | 0.13 | * | 5 |
| *Festuca pseudovina* | complex | grassland | 0.13 | * | 10 |
| *Festuca pratensis* | complex | grassland | 0.13 | * | 5 |
| *Capsella bursa-pastoris* | complex | fox burrow | 0.56 | *** | 32 |
| *Polygonum aviculare* | complex | fox burrow | 0.43 | *** | 22 |
| *Bromus tectorum* | complex | fox burrow | 0.33 | ** | 42 |
| *Chenopodium album* | complex | fox burrow | 0.26 | ** | 19 |
| *Bromus mollis* | complex | fox burrow | 0.26 | ** | 33 |
| *Torilis arvensis* | complex | fox burrow | 0.24 | ** | 12 |
| *Elymus repens* | complex | fox burrow | 0.22 | * | 29 |
| *Hordeum murinum* | complex | fox burrow | 0.20 | *** | 6 |
| *Onopordum acanthium* | complex | fox burrow | 0.20 | *** | 6 |
| *Tripleurospermum perforatum* | complex | fox burrow | 0.13 | * | 5 |
| *Carex praecox* | cleared | grassland | 0.55 | *** | 26 |
| *Salvia nemorosa* | cleared | grassland | 0.44 | *** | 23 |
| *Elymus hispidus* | cleared | grassland | 0.41 | *** | 33 |
| *Phlomis tuberosa* | cleared | grassland | 0.33 | *** | 10 |
| *Thymus glabrescens* | cleared | grassland | 0.24 | ** | 9 |
| *Euphorbia cyparissias* | cleared | grassland | 0.17 | ** | 9 |
| *Festuca rupicola* | cleared | grassland | 0.15 | * | 11 |
| *Fragaria viridis* | cleared | grassland | 0.10 | * | 3 |
| *Carduus acanthoides* | cleared | fox burrow | 0.30 | ** | 41 |
| *Cynodon dactylon* | cleared | fox burrow | 0.16 | ** | 9 |
| *Papaver rhoeas* | cleared | fox burrow | 0.15 | ** | 7 |
| *Fallopia convolvulus* | cleared | fox burrow | 0.14 | * | 7 |
| *Phragmites australis* | cleared | fox burrow | 0.13 | * | 12 |
